# Long-term effects and predictors of change of internet-delivered cognitive behavioural therapy on cardiac anxiety in patients with non-cardiac chest pain: a randomized controlled trial

**DOI:** 10.1186/s12888-024-05661-y

**Published:** 2024-03-19

**Authors:** Magda Eriksson-Liebon, Mats Westas, Peter Johansson, Ghassan Mourad

**Affiliations:** 1 Department of Health, Medicine and Caring Sciences, Linköping University, Linköping, Sweden; 2 Department of Emergency Medicine in Norrköping, and, Department of Biomedical and Clinical Sciences, Linköping University, Norrköping, Sweden; 3 Department of Internal Medicine in Norrköping, and, Department of Health, Medicine and Caring Sciences, Linköping University, Norrköping, Sweden

**Keywords:** Cardiac anxiety, Cognitive behavioural therapy, Health-related quality of life, Non-cardiac chest pain, Psychological distress

## Abstract

**Background:**

Approximately half of patients who seek care at Emergency Departments due to chest pain are diagnosed with Non-Cardiac Chest Pain (NCCP). Concerns for heart disease and misinterpretation of the symptoms increase cardiac anxiety and have a negative impact on patients' lives. Psychological interventions such as internet-delivered cognitive behavioral therapy (iCBT) are effective in treating psychological conditions such as anxiety, by helping patients to learn how to manage chest pain.

**Aims:**

To evaluate the effects of a nurse-led iCBT program on cardiac anxiety and secondary outcomes, as bodily sensations, depressive symptoms, health-related quality of life and chest pain frequency in patients with NCCP at 6- and 12-month follow-up, and to explore predictors that can have impact on the effects of the iCBT program on psychological distress.

**Methods:**

A longitudinal study of a Randomized Controlled Trial (RCT) evaluating the long-term effects of an iCBT program (*n* = 54) in patients with NCCP, compared to psychoeducation (*n* = 55). The primary outcome, cardiac anxiety was measured using the Cardiac Anxiety Questionnaire (CAQ), and the secondary outcomes were measured with The Body Sensations Questionnaire (BSQ), Patient Health Questionnaire-9 (PHQ-9), The EuroQol Visual Analog Scale (EQ-VAS) and a self-developed question to measure chest pain frequency. All measurements were performed before and after the intervention, and 3, 6 and 12 months after the intervention. Linear mixed model was used to test between-group differences in primary and secondary outcomes and multiple regression analysis was used to explore factors that may have an impact on the treatment effect of iCBT on cardiac anxiety.

**Results:**

A total of 85% (*n* = 93/109) participants completed the 12-month follow-up. Mixed model analysis showed no statistically significant interaction effect of time and group between the iCBT and psychoeducation groups regarding cardiac anxiety over the 12-month follow-up. However, there was a statistically significant interaction effect of time and group (*p* = .009) regarding chest pain frequency favouring the iCBT group. In addition, we found a group effect in health-related quality of life (*p* = .03) favouring the iCBT group. The regression analysis showed that higher avoidance scores at baseline were associated with improvement in cardiac anxiety at 12-month follow-up.

**Conclusions:**

Cardiac anxiety was reduced in patients with NCCP, but iCBT was not more effective than psychoeducation. Patients with a high tendency to avoid activities or situations that they believe could trigger cardiac symptoms may benefit more from psychological interventions targeting cardiac anxiety.

**Trial Registration:**

The trial was registered at ClinicalTrials.gov NCT03336112 on 08/11/2017.

## Contribution of the Paper

*What is already known*

- Almost half of all patients who seek care at emergency departments due to chest pain are diagnosed with non-cardiac chest pain.
- Recurrent non-cardiac chest pain leads to psychological distress and decreased quality of life, increased healthcare use, interruption of daily activities and absence from work.
- Cognitive behavioural therapy is an effective treatment for psychological distress and non-cardiac chest pain, helping patients with non-cardiac chest pain to get a correct and more realistic view on their chest pain and to learn how handle it.

*What this paper adds*

- Internet-delivered cognitive behavioural therapy reduces cardiac anxiety and improves health-related quality of life in patients with non-cardiac chest pain but is not more effective than psychoeducation.
- Higher levels of avoidance are associated with greater improvement in cardiac anxiety in the long term.

## Introduction

Chest pain is today one of the most common symptoms that often drives patients to primary or emergency care [1, 2]. However, in approximately half of the cases, chest pain is found to be of non-cardiac origin (i.e., non-cardiac chest pain (NCCP) [3]. NCCP, with lifetime prevalence between 20 and 40% [4–6], is a complex and multifaceted condition [6] with various origins, including gastrointestinal, musculoskeletal, pulmonary, as well as psychiatric factors [6, 7]. In addition, previous research show that the annual societal cost per patient with NCCP, is approximately €10,068 [8, 9]. Despite exclusion of cardiac reasons, many of these patients continue to worry about heart disease [10], avoid activities they consider harmful and seek medical help repeatedly as they have re-current chest pain episodes [11–14]. This situation leads to cardiac anxiety, which includes fear of heart-related sensations, increased attention to heart-related symptoms and avoidance of triggering activities [15], and impaired quality of life [11, 12]. Furthermore, cardiac anxiety has shown to create a vicious circle, leading to new chest pain episodes and thus more fear and anxiety, and increased healthcare use [16–19]. This suggests that patients with NCCP need help and support to evaluate the perception and management of their chest pain [20]. This can be achieved through cognitive behavioural therapy (CBT) which has strong support and has been shown to be effective in treating mild and moderate anxiety [21–24] and is also beneficial in treating NCCP [10, 25]. CBT is a collaborative and structured process that aims to support patients in evaluating the usefulness and accuracy of their thoughts and can be seen as an umbrella term including therapeutic interventions to treat various psychological problems [20, 26]. However, access to CBT is low due to a treatment demand gap [27, 28]. Therefore, internet-delivered CBT (iCBT) has increased in popularity since it does not differ from face-to-face CBT regarding the effects [29–32], requires less therapist involvement, can be given to more patients, is cheaper, and not time or location dependent [27]. However, there is a limited number [16, 33, 34] of studies examining the effects of iCBT on anxiety in patients with NCCP.

In our recent Randomized Controlled Trial (RCT) [10], we evaluated the short-term (up to 3 months) effects of a 5-week nurse-led iCBT-program compared to psychoeducation on cardiac anxiety and other patient-reported outcomes in patients with NCCP. We found no statistically significant interaction effect of time and group between the iCBT and psychoeducation groups, but within-group analysis showed improvement in cardiac anxiety at 3-month follow-up (*p* = *0.0*4) only in the iCBT group. Anyhow, long-term effects have only been reported by Thesen et al. [33, 34]. This is important since a Cochrane review [25] pointed out the need of RCTs for psychological interventions in patients with NCCP with long follow-up periods. In addition, it is important to find out which factors that may impact the effect of the iCBT treatment on cardiac anxiety in patients with NCCP. Such knowledge could help us understand which patients might benefit from iCBT. Therefore, the aim of this study was to evaluate the long-term effects (i.e., 6- and 12-month follow-up) of a nurse-led iCBT program on cardiac anxiety and other patient-reported outcomes in patients with NCCP, and to investigate factors that may have an impact on the effects of the iCBT program on these outcomes.

## Methods

The study design, participants, and procedures have already been reported by Mourad et al. [16] and therefore they are briefly described in this study.

### Design

A longitudinal randomized controlled design with 12-month follow-up was used. Due to the nature of the intervention being a CBT program, it was not feasible to keep patients unaware of their treatment group, and through that, masking was not possible.

### Study participants

To be included in the study, patients had to be 18 years or older, have at least two healthcare consultations due to NCCP (ICD-10 codes: R07.2, R07.3, R07.4, and Z03.4) in the last 6 months, suffer from cardiac anxiety (scoring at least 24 on the Cardiac Anxiety Questionnaire [CAQ]) [15], be able to speak and read Swedish, perform physical activity and have access to a computer or a tablet with Internet connection. Participants were recruited after discharge from four emergency departments in southeast Sweden between 2018 and 2020. Eligible participants were identified via registers and invited to participate and those who met the criteria and consented to participate were randomized into iCBT or psychoeducation using a randomization table provided by a statistician.

### The internet-based cognitive behavioural therapy program and the psychoeducation

In brief, patients in the intervention group received a 5-week/session nurse-led iCBT program comprising psychoeducation, mindfulness, exposure to physical activity, and weekly homework assignments with weekly feedback. Different approaches of CBT can be used to help patients modify their thoughts, behaviours and emotions. The aim of the iCBT program was to provide patients with knowledge and tools to decrease their cardiac anxiety and thereby to manage their chest pain.

The control group in the study was an active control group and received a 5-week psychoeducation program. The psychoeducation program was the same as for the iCBT group (containing information about NCCP, mindfulness and physical activity), but without homework assignments or feedback. The reasons for having an active control group are that patients with NCCP normally receive little attention for their problems and are usually not followed up in routine care because no physical causes can be found that can explain their chest pain. Being enrolled to treatment studies, e.g., iCBT in this study, after fulfilling the criteria for psychological distress and randomized to care as usual seems therefore unethical. It is also recommended that control groups in computerized CBT studies should receive some kind of attention, to know if the improvement is a specific effect of CBT or more generally [35].

### Data collection and measurements

Data in this long-term follow-up were collected online at baseline and 5 weeks after (postintervention), and 3, 6, and 12 months postintervention. Medical records were used to provide medical data, but the rest of the data were self-reported by participants. For data used to determine the factors associated with change in cardiac anxiety at 12-month follow-up, see Table 1.

**Table 1:** Demographic data, medical information and outcome variables in study patients at baseline

	**iCBT (*n* = 54)**	**Psychoeducation** **(*n* = 55)**	***p***
**Age year (mean ± SD)**	54.3 ± 16.5	56.8 ± 15.5	.416
**Sex n (%)**			.639
Females	32 (59)	35 (64)	
Males	22 (41)	20 (36)	
**Marital status, n (%)**			.081
Married/ Co-habitants	45 (83)	38 (69)	
Single	9 (17)	17 (31)	
**Economic situation n (%)**			.453
Good	47 (87)	45 (82)	
Problematic	7 (13)	10 (18)	
**Educational level n (%)**			.892
Elementary/ High school	30 (56)	33 (60)	
University	24 (44)	22 (40)	
**Occupational status n (%)**			.776
Working	26 (48)	28 (51)	
No working	28 (52)	27 (49)	
**Smoking n (%)**			.970
None/previous smoker	49 (91)	48 (87)	
Smoker	5 (9)	7 (13)	
**Alcohol consumption n (%)**			.763
No drinking (0–4 glasses)	83 (45)	85 (47)	
Drinking (> 5 glasses)	17 (4)	18 (8)	
**Charlson Comorbidity Index (mean ± SD)**	2.2 (2.4)	2.5 (2.1)	.566
**Previous heart disease n (%)**	18 (33)	15 (27)	.496
**Psychotherapy n (%)**	8 (15)	5 (9)	.357
**Psychotropic treatment n (%)**	23 (43)	25 (45)	.763
**Cardiac Anxiety at baseline (CAQ, mean ± SD)**	36 (8)	36 (9)	.959
CAQ Fear at baseline (mean ± SD)	19 (4)	19 (5)	.901
CAQ Avoidance at baseline (mean ± SD)	8 (4)	8 (4)	.735
CAQ Attention at baseline (mean ± SD)	9 (4)	9 (3)	.460
**Fear of bodily sensations at baseline (BSQ, mean ± SD)**	43 (14)	42 (12)	.621
**Depressive symptoms at baseline (PHQ9, mean ± SD)**	8 (6)	8 (6)	.755
**Health-related quality of life at baseline (EQ-VAS, mean ± SD)**	59 (20)	66 (17)	.031
**Chest pain frequency at baseline (mean ± SD)**	15 (13)	9 (11)	.006

*Abbreviations: CAQ* Cardiac Anxiety Questionnaire, *BSQ* The Body Sensations Questionnaire, *PHQ-9* Patient Health Questionnaire-9, *(EQ-VAS)* The EuroQol Visual Analog Scale

#### Primary outcome

Cardiac anxiety was measured using CAQ, which is an 18-item questionnaire. Higher scores indicate more severe cardiac anxiety. The CAQ has shown good reliability and validity [15]. The Cronbach α coefficients were 0.79 to 0.89 for the iCBT and the psychoeducation groups in this study. The CAQ consists of three subscales: fear, avoidance, and heart-focused attention [15].

#### Secondary outcome

Fear of bodily sensations (e.g., heart palpitations and fainting) were measured with The Body Sensations Questionnaire (BSQ) [36]. Higher scores indicate higher fear of bodily sensations. This 17-item questionnaire has demonstrated good psychometric properties. In this study, the Cronbach α coefficients were 0.92 to 0.94. The Patient Health Questionnaire-9 (PHQ-9) consisting of 9 items with score range between 0 and 27, was used to measure depressive symptoms. Scores over 10 indicate at least moderate depressive symptoms. The PHQ is a valid and reliable measurement [37], with Cronbach α coefficients of 0.87 to 0.89 in this study. Health-related quality of life was measured with the EuroQol Visual Analog Scale (EQ-VAS). The scale ranges between 0 – 100, where 0 is the worst imaginable health state and 100 is the best imaginable health state [38]. Chest pain frequency was measured with a question that was developed by the authors: During the last month, how often have you experienced NCCP?

### Statistical analyses

We used frequencies, percentages, mean values, and SDs to describe the background variables. For comparison between groups, Chi-square test or Student’s t-test were used depending on the level of the data. Power analysis was performed based on results from our previous pilot study [34] and indicated a need of 106 participants divided into the two arms, i.e., intervention and control, to reach a 20% improvement (approximately an effect size of 0.5) in cardiac anxiety (95% CI, 80% power). The sample size in this study is comparable to similar iCBT-studies [39].

For the long-term analyses, linear mixed models were performed to compare changes between the iCBT and psychoeducation groups regarding cardiac anxiety, fear of bodily sensations, depressive symptoms, health-related quality of life, and chest pain frequency. All analysis were done on original data with no imputation using linear mixed models, since linear mixed model is regarded as a robust method, particularly in handling unbalanced and missing data [40]. Intention-to-treat analysis was applied to minimize the risks of bias related to differences in groups caused of non-adherence or missing data [41]. However, all long-term analyses were performed on original data as we had little missing data (15% at both 6- and 12-month follow-up). Chest pain frequency data were not normally distributed at baseline. However, mixed model analysis is considered as a robust method that can handle such data. Anyhow, we also estimated a standardized value (z-value) for chest pain frequency, but these results did not differ from the mixed model results that was based on original data. This is therefore not presented in the results.

The effect size of the intervention was measured with Cohen’s *d,* which categorizes effects as trivial (< 0.20), small (0.20 to 0.49), moderate (0.50 to 0.79), and large (≥ 0.80) [42]. As CAQ lacks an established cut-off score indicating prevalence or severity of cardiac anxiety, a reliable change index score was calculated in the main study [16] to assess improvement in cardiac anxiety. According to Christensen and Mendoza, [43] this index was calculated by dividing the SD score of 8.6 by and the Cronbach α coefficient score of 0.79 at baseline in our groups. Based on these values, a reliable change was considered at a score of 11 points per participant. Differences between groups in the number of patients with a change score ≥ 11 at all measurement points (i.e., 5 weeks, and 3, 6, and 12 months) were calculated with the chi-square test.

To explore factors that may have an impact on the treatment effect of iCBT on cardiac anxiety, changes in cardiac anxiety (CAQ score) between baseline and 12-month follow-up were used as the dependent variable in a multiple regression analysis. Since we do not know which factors can affect the treatment effect of iCBT on cardiac anxiety, we used an exploratory approach and tested all variables from Table 1. A two-step algorithm was used to find possible exploratory variables. The first step consisted of a univariate analysis of variance to find variables to be included in a multiple regression model. In that analysis we explored the relationship between demographic and medical variables and the change score in CAQ between baseline and 12-month follow-up. In the univariate analysis of variance, we also included baseline scores of the CAQ total and the subscales (fear, avoidance and heart-focused attention), BSQ, PHQ-9, EQ-VAS and chest pain frequency. Variables with *p* < 0.20 were used as exploratory variables in the multiple regression analysis. According to Altman, [44] selected variables should be based on a lax criterion, e.g. *p* < 0.20 or higher, due to unforeseen ways in which the variables may contribute to a multiple regression model, due to complex relationships between the variables. These variables (i.e., psychotropic treatment, CAQ total baseline, CAQ avoidance baseline and CAQ attention baseline) were entered simultaneously into the multiple regression analysis and non-significant variables (*p* > 0.05) were deleted from the multiple regression analysis and re-analyzed until all variables in the final model were found to be statistically significant. The final model met the assumption for not containing multicollinearity variables (i.e., violation inflation factor < 10). Only original data were used, and missing data were not imputed in the multiple regression analysis. The IBM SPSS version 28.0 was used for data analysis and *p* < 0.05 was considered as statistically significant.

## Results

**Fig. 1:**
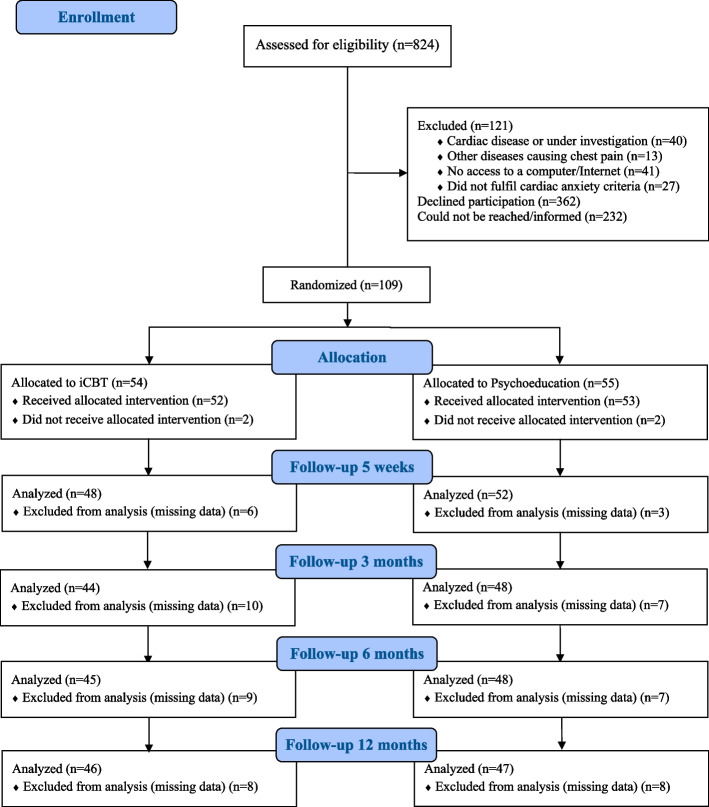
The CONSORT flowchart

In total, 824 patients were assessed for eligibility and invited to participate in the study, see Fig. 1. Out of these, 230 patients (28%) were possible to assess, and 109 (47%) fulfilled the inclusion criteria and consented to participate and were thereby randomized into 5 weeks nurse-led iCBT (54/109, 49.5%) or psychoeducation (55/109, 50.4%). The demographic data for the iCBT and psychoeducation groups are presented in Table 1. The mean age was 56 years (SD 16) and most of participants were women (62%, *n* = 67), not working (51%, *n* = 55), and married or in a relationship (76%, *n* = 83). The majority (70%, *n* = 76) had no previous heart disease. Regarding treatment for psychological distress, 88% (*n* = 96) had no experience of psychotherapy, and 56% (*n* = 61) had not been prescribed psychotropic treatment. No significant differences in the characteristics or medical data were found at baseline between patients randomized to iCBT or psychoeducation except for health-related quality of life (*p* = 0.031) and chest pain frequency (*p* = 0.006).

Of the 109 patients included in the study, a total of 15% (*n* = 16); iCBT *n* = 8 and psychoeducation *n* = 8) had missing data at the 12-month follow-up, as patients did not complete the questionnaires. Reasons for drop-out were not reported. The mean age for those who dropped out in the iCBT group was 51 years (SD 19), and the majority of participants were women (75%, *n* = 6). In the psychoeducation group, the participants who dropped out were 55 years old (SD 21), and most of them were women (63%, *n* = 5). The patients who continued their participation in the iCBT group had a mean age of 55 years (SD 16) and were mostly women (57%, *n* = 26). In the psychoeducation group, the corresponding numbers were 57 years (SD 15) and mostly women (64%, *n* = 30). A drop-out analysis did not reveal any statistically significant differences between those who continued or terminated their participation.

### Long-term effects of iCBT

**Fig. 2:**
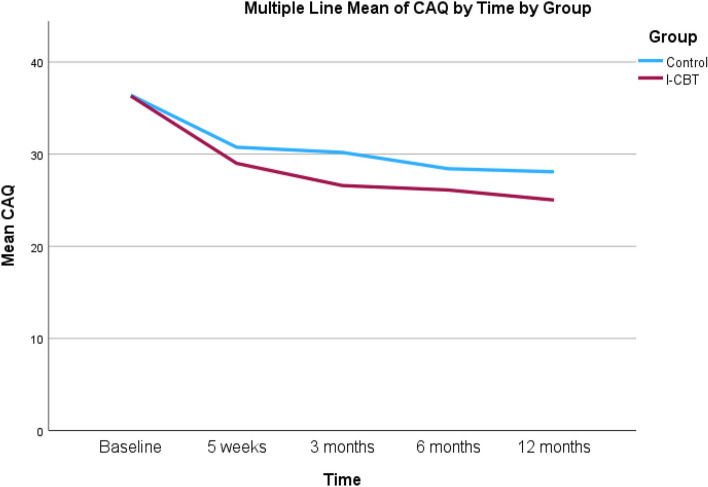
Changes in cardiac anxiety over time between iCBT and psychoeducation group

Regarding the primary outcome, Cardiac anxiety, the mixed model analysis showed no statistically significant interaction effect of time and group (*p* = 0.256; Cohen’s *d* = 0.31) between the iCBT and psychoeducation groups over the 12-month follow-up (Table 2). Within group analyses showed statistically significant improved cardiac anxiety during the 12-moths follow-up (*p* < 0.001) in both iCBT and psychoeducation groups (Table 3). Figure 2 displays changes in cardiac anxiety over 12-month follow-up between the groups.

**Table 2:** Mixed model analysis of the effect of Internet-delivered cognitive behavioural therapy (iCBT) compared to psychoeducation on cardiac anxiety and secondary outcomes, presented in estimated marginal means

	**Time effect**			**Group effect**		**Interaction effect**			**Effect size Cohens´ *d***
Variables	Mean	*P*-value^a^	iCBT	Psychoeducation	*P*-value	iCBT	Psychoeducation	*P*-value^a^	
**CAQ**^b^			29.4	30.8	.959				0.31
Baseline	36.4	-				36.3	36.4	-	
5 weeks	30.3	** < .001**				29.8	30.8	.493	
3 months	29.0	** < .001**				28.1	30.0	.312	
6 months	28.1	** < .001**				27.1	29.1	.273	
12 months	26.8	** < .001**				25.8	27.8	.256	
**BSQ**^c^			38.2	38.5	.618				0.40
Baseline	42.7	-				43.3	42.1	-	
5 weeks	38.0	** < .001**				37.0	39.1	.092	
3 months	37.5	** < .001**				35.7	37.3	.664	
6 months	36.8	** < .001**				37.6	35.9	.872	
12 months	36.8	** < .001**				35.3	38.3	.086	
**PHQ-9**^d^			6.6	6.3	.752				0.07
Baseline	7.8	-				8.0	7.6	-	
5 weeks	7.0	.515				7.6	6.4	.324	
3 months	5.9	.**005**				6.0	5.8	.901	
6 months	6.2	**.003**				6.1	6.3	.476	
12 months	5.4	**< .001**				5.3	5.5	.546	
**EQ-VAS**^e^			61.1	65.7	**.030**				0.27
Baseline	62.5	-				58.5	66.4	-	
5 weeks	61.2	.579				60.0	62.3	.139	
3 months	61.9	.304				62.2	61.6	.094	
6 months	64.9	.629				60.0	69.9	.642	
12 months	66.5	**.027**				64.9	68.2	.258	
**Chest pain frequency**			10.6	6.7	**.005**				0.48
Baseline	12.0	-				15.1	8.8	-	
5 weeks	11.1	.613				14.2	8.1	.923	
3 months	8.1	**.003**				9.4	6.7	.164	
6 months	6.8	**< .001**				8.7	4.9	.247	
12 months	5.2	**< .001**				5.6	4.8	**.009**	

^a^In comparison with baseline

^b^*CAQ* Cardiac Anxiety Questionnaire

^c^*BSQ* Body Sensations Questionnaire

^d^*PHQ−9* Patient Health Questionnaire−9

^e^*EQ−VAS* EuroQol Visual Analogue Scale

**Table 3:** Within group changes (Estimates of Fixed Effects from Linear Mixed Model Analysis) over the 12-month follow-up in the iCBT and psychoeducation groups

		**iCBT group**							**Psychoeducation group**						
Variable (Instrument)	Time	Mean	Std. Error	Estimate	Sig	95% Confidence Interval Low. Bound Upp. Bound		Nr. of Subjects	Mean	Std. Error	Estimate	Sig	95% Confidence Interval Low. Bound Upp. Bound		Nr. of Subjects
**Cardiac anxiety** (CAQ)	0 Weeks	36.32	1.109	.00^a^				54	36.40	1.214	.00^a^				55
	5 Weeks	29.81	1.229	-6.504	< .001	-8.641	-4.367	48	30.83	1.387	-5.569	< .001	-7.377	-3.762	52
	3 Months	27.98	1.467	-8.339	< .001	-11.043	-5.634	44	30.03	1.644	-6.373	< .001	-8.602	-4.145	48
	6 Months	26.82	1.400	-9.472	< .001	-12.133	-6.811	45	29.14	1.637	-7.260	< .001	-9.444	-5.076	48
	12 Months	25.58	1.322	-10.736	< .001	-13.383	-8.089	46	27.84	1.553	-8.556	< .001	-10.883	-6.230	47
**Fear of bodily sensations** (BSQ)	0 Weeks	43.33	1.943	.00^a^				54	42.07	1.607	.00^a^				55
	5 Weeks	36.86	1.509	-6.474	< .001	-9.427	-3.521	48	39.10	1.703	-2.975	.023	-5.528	-.423	52
	3 Months	37.76	1.756	-5.577	< .001	-8.078	-3.075	44	37.30	1.750	-4.777	.003	-7.907	-1.648	48
	6 Months	37.72	1.944	-5.610	< .001	-8.829	-2.391	45	35.89	1.606	-6.181	< .001	-9.323	-3.040	48
	12 Months	35.43	1.665	-7.908	< .001	-10.907	-4.909	46	38.27	1.870	-3.803	.061	-7.785	.180	47
**Depressive symptoms** (PHQ-9)	0 Weeks	8.00	.768	.00^a^				54	7.64	.853	.00^a^				55
	5 Weeks	7.63	.739	-.374	.485	-1.444	.695	48	6.42	.834	-1.219	.060	-2.491	.053	52
	3 Months	6.29	.746	-1.711	< .001	-2.595	-.827	44	5.77	.784	-1.867	.024	-3.480	-.255	48
	6 Months	6.08	.722	-1.925	.002	-3.133	-.717	45	6.31	.722	-1.324	.043	-2.606	-.042	48
	12 Months	5.33	.579	-2.667	< .001	-3.805	-1.529	46	5.53	.779	-2.104	.005	-3.534	-.673	47
**Health-related quality of life** (EQ-VAS)	0 Weeks	58.54	2.760	.00^a^				54	66.38	2.266	.00^a^				55
	5 Weeks	60.18	2.863	1.640	.430	-2.498	5.779	48	62.18	2.908	-4.204	.181	-10.419	2.011	52
	3 Months	62.70	3.209	4.166	.235	-2.788	11.120	44	61.84	3.500	-4.542	.211	-11.742	2.657	48
	6 Months	59.63	3.082	1.088	.684	-4.255	6.432	45	69.48	2.613	3.094	.359	-3.620	9.808	48
	12 Months	64.66	2.893	6.121	.012	1.410	10.833	46	67.95	2.754	1.572	.620	-4.749	7.893	47
**Chest pain frequency** (Self-developed question)	0 Weeks	15.15	1.704	.00^a^				54	8.99	1.414	.00^a^				54
	5 Weeks	13.81	2.474	-1.335	.597	-6.373	3.704	47	8.15	1.436	-.845	.473	-3.193	1.503	52
	3 Months	9.25	2.000	-5.899	.005	-9.980	-1.817	44	6.84	1.602	-2.156	.199	-5.490	1.177	48
	6 Months	8.90	1.636	-6.240	< .001	-9.660	-2.819	45	4.92	.976	-4.085	.004	-6.808	-1.361	48
	12 Months	5.82	1.201	-9.329	< .001	-12.613	-6.045	46	4.79	1.088	-4.207	.002	-6.766	-1.647	47

*Abbreviations:* *CAQ* Cardiac Anxiety Questionnaire, *BSQ* Body Sensations Questionnaire, *PHQ-9* Patient Health Questionnaire-9, *EQ-VAS* EuroQol Visual Analogue Scale

^*a*^Used as time reference

Looking to the positive reliable change score at 12-month follow-up (i.e., a decrease in cardiac anxiety score of ≥ 11), in the iCBT group, 46% (21/46) of patients had a positive reliable change score. The corresponding number was 34% (16/47) in the psychoeducation group. The difference between the iCBT and psychoeducation groups was statistically not significant (*p* = 0.25). One participant had a negative reliable change score (i.e., an increase in cardiac anxiety score of ≥ 11) at 6-month follow-up and no one had a negative reliable change score at 12-month follow-up.

**Fig. 3:**
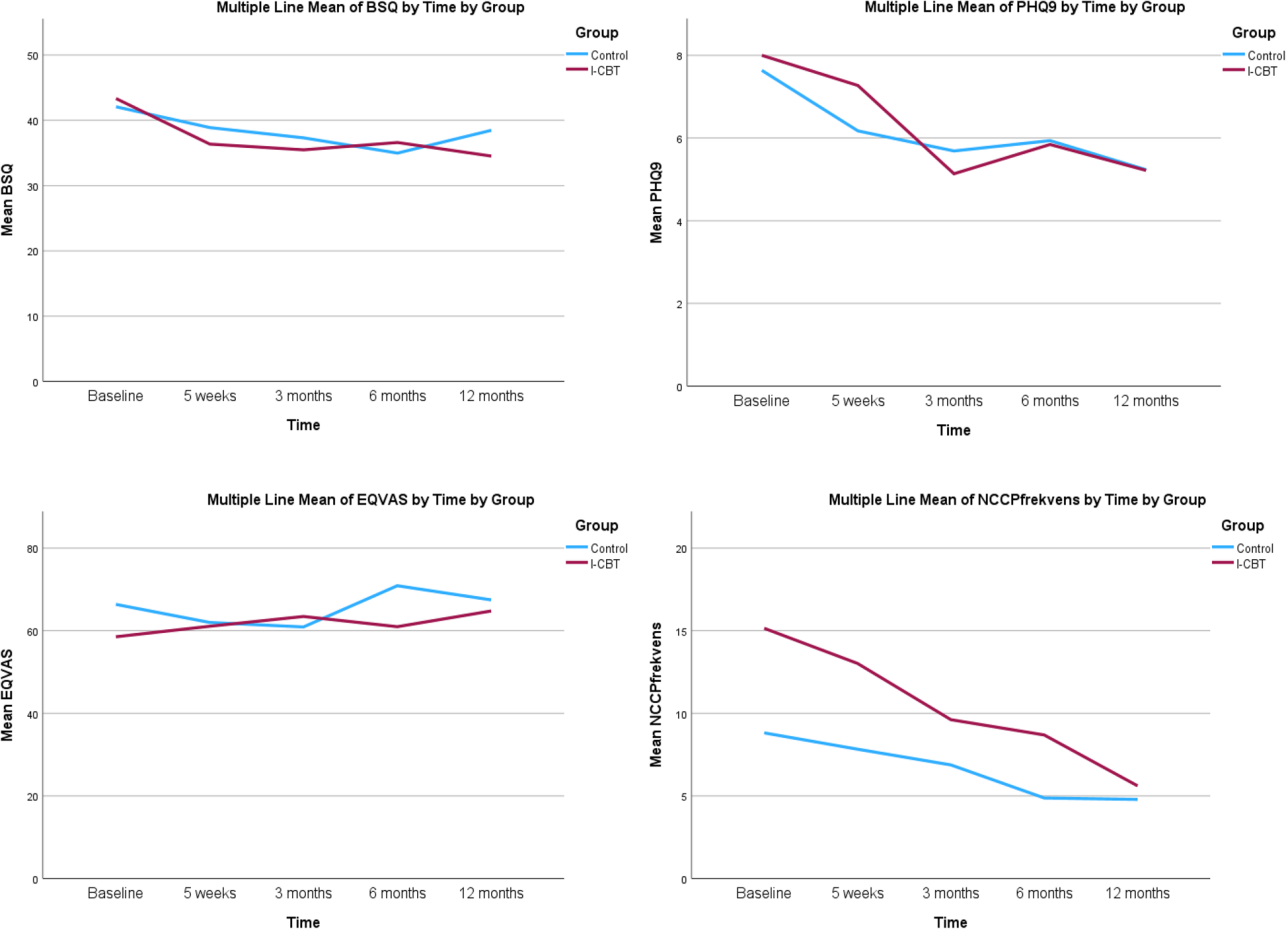
Changes in bodily sensations, depressive symptoms, heart-related quality of life, and non-cardiac chest pain frequency over time between iCBT and psychoeducation group

In the secondary outcomes, we found no statistically significant interaction effect of time and group between the iCBT and psychoeducation groups regarding fear of bodily sensations, depressive symptoms, and health-related quality of life during the 12-month follow-up (see Table 2). However, the analysis showed a statistically significant interaction effect of time and group regarding chest pain frequency (*p* = 0.009; Cohen’s *d* = 0.48) in favour of the iCBT group at the 12-month follow-up. We also found a group effect in health-related quality of life (*p* = 0.03; Cohen’s *d* = 0.27) favouring the iCBT group. Long-term changes in bodily sensations, depressive symptoms, health-related quality of life, and non-cardiac chest pain frequency between iCBT and psychoeducation groups are displayed in Fig. 3. These changes are also presented in detail in the Table 3.

### Factors associated with change in cardiac anxiety at 12-month follow-up

From the univariate analysis of variance, we found four exploratory variables to include in the multiple regression analysis. These were psychotropic treatment (*p* = 0.065), CAQ total baseline (*p* = 0.035), CAQ avoidance baseline (*p* = 0.008), and CAQ attention baseline (*p* = 0.153). Variables that were excluded were group (*p* = 0.410), age (*p* = 0.686), gender (*p* = 0.646), marital status (*p* = 0.805), economy status (*p* = 0.731), educational level (*p* = 0.331), occupational status (*p* = 0.564), smoking (*p* = 0.578), alcohol consumption (*p* = 0.439), Charlson Comorbidity Index (*p* = 0.922), previous heart disease (*p* = 0.950), psychotherapy (*p* = 0.991), CAQ fear baseline (*p* = 0.729), BSQ baseline (*p* = 0.417), PHQ-9 baseline (*p* = 0.739), EQ-VAS baseline (*p* = 0.459), and chest pain frequency baseline (*p* = 0.735).

All four statistically significant variables were entered simultaneously into the multiple regression model, and only CAQ avoidance at baseline was statistically significant (beta = 0.355, *p* = 0.034) implying that the more avoidance at baseline, the greater the likelihood of reduced cardiac anxiety at 12 months.

## Discussion

In this longitudinal study we evaluated the effects of an iCBT program targeting cardiac anxiety compared to psychoeducation. The results show that iCBT was not superior to psychoeducation in decreasing cardiac anxiety at 12-month follow-up. We also found that higher scores on the subscale CAQ avoidance were associated with greater decrease in cardiac anxiety at 12-month follow-up.

To our knowledge, only one longitudinal study [33] has been conducted in patients with NCCP and shown positive effects on cardiac anxiety in favor of iCBT. In that study, iCBT was compared with treatment as usual, while our study used an active control, i.e., psychoeducation. We believe this may explain why they, but not us, were able to establish iCBT as a superior intervention. Since both our studies used the same primary outcome questionnaire (i.e., CAQ) it is also possible to compare the improvement in cardiac anxiety. Their patients decreased from 23.2 to 13.8 in the CAQ total score, between baseline and 12-month follow-up, while the scores in our study decreased from 36.3 to 25.6. Thus, both groups decreased about 10 points. Furthermore, the improvement in the iCBT group was statistically significant at 12-month follow-up compared to baseline, which also was the case in the psychoeducation group as they received the same psychoeducational program but without homework assignments and feedback from the therapist. One may criticize the choice of using an active control group instead of using waiting list control. However, it is known that iCBT is superior to waiting list, and waiting list control studies do not provide insights if the improvement in the primary outcome is due to the iCBT intervention or other factors such as increased activity or attention to condition [45–47]. Therefore, we chose to have an attention control group, i.e., psychoeducation, in order to determine whether or not the effects are linked to the intervention.

Although, we could not establish significant differences in cardiac anxiety between the groups, a total of 46% of the iCBT group compared to 34% of the psychoeducation group had a positive reliable change score with ≥ 11 points on CAQ at 12-month follow-up. We also found statistically significant improvement regarding chest pain frequency (*p* = 0.009) favouring the iCBT group. However, this result should be interpreted with caution as chest pain frequency was measured with a self-developed question, and that we despite randomization had an imbalance in chest pain frequency scores at baseline between the groups. Furthermore, comparable to Thesen et al. [33], our iCBT group showed a statistically significant improvement in health-related quality of life (*p* = *0.0*3) at 12-month follow-up. Lesson learned, from our and the study by Thesen et al. [33], suggest that iCBT can be used to decrease cardiac anxiety and improve health-related quality of life in patients with NCCP. However, from our results, we cannot conclude that iCBT is better than psychoeducation in decreasing cardiac anxiety in these patients, but a meta-analysis in patients with chronic conditions, found that guided and unguided iCBT interventions had similar effects on anxiety symptoms [47]. The unguided intervention can to some extent be compared to our active control group, which received psychoeducation about chest pain, cardiac anxiety, mindfulness, physical activity and exposure to physical activity. Thus, guided or unguided psychoeducation can be seen as an important part of iCBT programs targeting cardiac anxiety in patients with NCCP.

We found that higher levels of CAQ avoidance were associated with greater improvement in cardiac anxiety between 12-month follow-up and baseline. This is consistent with results from other studies [35, 45, 48] showing that patients with high severity of psychological distress at baseline, are more prone to benefit from the iCBT treatment. The avoidance subscale in the CAQ refers to the individual’s tendency to avoid activities or situations that may trigger or exacerbate their cardiac symptoms [15]. Avoidance in patients with NCCP as well as cardiac diseases seems to be of importance to take into consideration since, Van Beek et al. [49] using CAQ in patients with myocardial infarction reported that higher avoidance scores 4 months after discharge were associated with increased risk of major adverse cardiac events. A possible mechanism could be increased physical inactivity due to fear of provoking cardiac symptoms. A study by Schmitz et al. [50] showed that more avoidance at baseline in patients undergoing psycho-cardiological rehabilitation negatively impacted future physical activity levels. To make this issue more complex, Castonguay et al. [51] reported that physical activity has a protective effect on NCCP-related disability (i.e., social, family, work and physical activity). This highlights the relevance of cardiac anxiety, and avoidance in particular, when designing and performing treatments for patients with NCCP. Adapting treatment to patients' individual needs and preferences can improve their adherence to treatment. When patients actively participate in shared decision-making processes concerning their treatment and when their choices regarding treatment options are respected, it promotes a patient-centered approach to care, which can increase patient participation in treatments (i.e., the iCBT program).

### Strengths and limitations

A total of 16 patients (15%) had missing data at the 12-month follow, which can be seen as a low drop-out rate in this type of longitudinal study. We therefore ran our mixed model analysis based on original data. One limitation is that we did not follow changes in the patients’ health status. Another limitation is that we did not collect data on healthcare use. This may have impacted how patients rated their cardiac anxiety in this study. The length of the intervention may be seen as another limitation. We are aware that the common duration for iCBT is 8–12 weeks. However, in this study we intended to evaluate if a shorter iCBT program could be effective in decreasing cardiac anxiety in patients with NCCP as one previous study by Jonsbu et al. [13] showed that a three-session CBT intervention was effective in decreasing avoidance of physical activity. However, the intervention was not internet-based.

## Conclusions

In this longitudinal follow-up of a RCT, where iCBT was compared with psychoeducation, cardiac anxiety was reduced. However, iCBT was not more effective than psychoeducation in reducing cardiac anxiety. Patients with a high tendency to avoid activities or situations that they believe could trigger cardiac symptoms may benefit more from psychological interventions targeting cardiac anxiety to increase their physical activity and thus reduce psychological distress.

## Data Availability

The datasets used and/or analyzed during the current study available from the corresponding author on reasonable request.

## Abbreviations

BSQ: The Body Sensations Questionnaire
CAQ: Cardiac Anxiety Questionnaire
EQ-VAS: The EuroQol Visual Analog Scale
iCBT: Internet-based Cognitive Behavioural Therapy
NCCP: Non-Cardiac Chest Pain
PHQ-9: Patient Health Questionnaire-9
RCT: Randomized Controlled Trial
